# Single Cell in a Gravity Field

**DOI:** 10.3390/life12101601

**Published:** 2022-10-14

**Authors:** Irina V. Ogneva

**Affiliations:** Cell Biophysics Laboratory, State Scientific Center of the Russian Federation Institute of Biomedical Problems of the Russian Academy of Sciences, 76a, Khoroshevskoyoe Shosse, 123007 Moscow, Russia; iogneva@yandex.ru; Tel.: +7-(499)-195-63-98

**Keywords:** cellular mechanoreception, cellular mechanotransduction, space flight, microgravity, hypergravity, early embryo, oocyte, spermatozoon

## Abstract

The exploration of deep space or other bodies of the solar system, associated with a long stay in microgravity or altered gravity, requires the development of fundamentally new methods of protecting the human body. Most of the negative changes in micro- or hypergravity occur at the cellular level; however, the mechanism of reception of the altered gravity and transduction of this signal, leading to the formation of an adaptive pattern of the cell, is still poorly understood. At the same time, most of the negative changes that occur in early embryos when the force of gravity changes almost disappear by the time the new organism is born. This review is devoted to the responses of early embryos and stem cells, as well as terminally differentiated germ cells, to changes in gravity. An attempt was made to generalize the data presented in the literature and propose a possible unified mechanism for the reception by a single cell of an increase and decrease in gravity based on various deformations of the cortical cytoskeleton.

## 1. Introduction

Gravity is one of the physical factors under which life appeared and developed on Earth. Remaining permanent, this force has a significant impact on the ontogenesis of all species and on some aspects of human life. Therefore, leaving the normal environment and staying for a long time, for example, in weightlessness, can lead to negative consequences for human health and performance, which significantly reduces the possibility of deep space exploration.

Thus, under space flight conditions, atrophic changes occur in the muscular system, especially in the postural muscles [1,2], and the skeletal system, which, after returning to gravity, prevents maintaining one’s posture and reduces performance [3,4,5]. The fluid shift in the cranial direction in weightlessness leads to a change in the work of the heart and the cardiovascular system [6,7,8,9]. The negative impact of microgravity on the nervous and other systems of the body is also well known [10,11]. The countermeasure system of possible changes is based on the modulation of the diet and, first of all, on the system of physical training [12,13].

However, negative changes in various systems under conditions of weightlessness and/or the subsequent return to gravity occur, including at the cellular level: in muscle fibers [14,15,16], in cardiomyocytes [15,16,17,18], in neurons [19,20], osteocytes [21], etc. Therefore, it can be assumed that the development of perspective methods for protecting the body during long-term space flight should be based mainly on the cellular level.

Today, there is no doubt that with a change in external mechanical stress in single cells of various types and levels of differentiation, an adequate cellular response is formed in the form of a pattern of structural proteins, changes in metabolism, and gene expression. However, the mechanism of gravitational field reception and mechanotransduction pathways for the formation of an adaptive response of single cells are poorly understood.

The development of any organism begins with a single, fully totipotent cell: a zygote, which is formed by the fusion of two terminally differentiated cells—a sperm and an oocyte. It is extremely interesting that already at the zygote stage, various organisms start the mechanism of protection from the negative impact of weightlessness, which leads to the birth of normal offspring and, in fact, the preservation of the species. Thus, when modeling weightlessness in eight-cell embryos of *Xenopus laevis*, the orientation of cell division changed, and a shift in the position of the blastocoel towards the center and an increase in the thickness of its cell layer were observed. However, despite all the changes in early embryogenesis, tadpoles at the feeding stage were indistinguishable from controls [22]. Successful coupling of vertebrates using the example of Medaka fish (4 individuals during a 15-day space flight aboard the shuttle space mission) was carried out in 1994, and, despite some difficulties in mating under weightless conditions, the fish laid 43 eggs, of which 8 fry appeared while still in space, and 30 within 3 days of landing. The fry hatched in space had a normal number of germ cells, and two of them also gave birth to healthy offspring on Earth [23]. At the same time, the *hir* mutant was obtained in this fish species, using which it was shown that the YAP protein, localized in the nucleus, is involved in the formation of the 3D body shape [24]. Additionally, in Japanese quail embryos, most of the disorders that occur at early stages of development under weightless conditions are leveled by later periods and/or in the early postnatal period [25]. We managed to obtain three generations of the fruit fly *Drosophila melanogaster* under the conditions of a 44.5-day space flight of the FOTON-M4 satellite (July–September 2014, Russia), which had no developmental deviations and retained fertility on Earth and during the subsequent flight to the ISS (October–November 2014), where the next two generations were obtained [26].

All this gives basis to the belief that a detailed understanding of the mechanism of plasticity realized in the earliest embryos can be useful for the development of new approaches to the prevention of disturbances caused by space flight factors. However, a number of difficulties in working with germ cells, zygotes, and early embryos under conditions of both real and simulated microgravity and hypergravity determine the currently limited pool of experimental data presented in this review.

## 2. Toti- and Pluripotent Cells

Studies of various stem cells in real or simulated microgravity are most often carried out in cultures, which has its limitations associated with the organization of the experiment. In particular, it is necessary to take into account the shear stresses that arise in the medium when using, for example, various rotating systems. In addition, the influence of various factors produced in the environment by the cells themselves can also modulate the gravitational response, although this is less true for early embryos.

### 2.1. Early Embryos

A change in external mechanical stress leads to significant changes in the structural and functional organization of zygotes and early embryos.

Eggs of the sprat frog *Rana temporaria* were cultivated for 2.5 h immediately after fertilization on a clinostat, and it was shown that under these conditions, the percentage of zygotes with an irregular distribution of cortical pigment and eccentric division lines increases [27]. Clinostating of fertilized eggs of the frog *Xenopus laevis* leads to a shift in the furrow of the third division in the direction of the equatorial plane, while hypergravity at the level of 15 *g* and *30* g for 4 min leads to significant variations in twinning, which the authors associated with the heterogeneity of the viscous cytoplasm of eggs [28].

In the early embryogenesis of a shellfish under microgravity conditions, asymmetric distribution of microtubules is observed, which affects the patterns of cleavage during morphogenesis [29], which can lead to morphological developmental anomalies, but possibly not of all organs. Thus, in the larvae of the newt, after 15 days of exposure to space flight conditions (IML-2 mission), the dimensions of the labyrinth were comparable to those in the control [30]. However, exposure to hypergravity affects a similar organ in Aplysia larvae: in individuals grown on a centrifuge with rotation from 1 *g* to 5 *g*, a decrease in statolith was observed with increasing g [30].

For mouse gametes, it was possible to carry out a full cycle of IVF under simulated weightlessness. Before fertilization, spermatozoa were cultured on a clinostat for 2 h, then added to oocytes, and exposure was started again under simulated microgravity. The authors of the study showed that after 6 h of cultivation in simulated weightlessness, zygotes are observed. After 12 h, two-cell embryos are observed, and after 96 h, mature blastocysts are observed. Embryos were implanted in females at the two-cell stage and at the blastocyst stage, successfully developed, were born alive, and were fertile [31].

However, if the exposure of mouse embryos was started at the zygote stage and cultured for 9 h in simulated microgravity, then these zygotes stopped at the pronuclear nondisjunction stage, apparently as a result of microtubule structure disturbance [32]. According to the authors, this may be due to changes in the expression of long non-coding RNAs (lncRNAs), although their role in pronuclear migration is still poorly understood. In particular, a decrease in the content of lnc007956, which binds both alpha-actinin4 (actin-binding proteins, a component of microfilaments) and beta-tubulin (subunit of Tubb4b), can disrupt the interaction of cytoskeletal components and, thus, prevent pronucleus divergence [32].

As part of the SJ-10 experiment (launch 6 April 2016, China), 3400 two-cell mouse embryos (that is, after the first zygote cleavage) were placed in the equipment 12 h before the launch. Some of the embryos were monitored using time-lapse; the rest were recorded after 64 h in flight. Video data obtained by time-lapse microscopy indicate that two-cell embryos cultured in space went through key stages of preimplantation development, including cleavage, compaction, cavitation, and blastocyst formation. However, developmental defects were observed in fixed embryos, in particular, changes in the spatial patterns of expression of the intracellular mass marker Oct4 and trophectoderm Cdx2 [33]. In addition, the methylome analysis of individual blastocysts showed that under space flight conditions, there is a total decrease in the level of methylation, and regulatory regions of the genome (promoters, untranslated regions, introns, and intergenic spaces) turn out to be hypomethylated, among other things. This effect, according to the authors of the study, is associated to a greater extent with the action of ionizing radiation in space flight, as evidenced by ground-based studies [33].

### 2.2. Embryonic Stem Cells (ESC)

Cultivation of mouse ESCs in simulated microgravity on a 3D clinostat for up to 7 days leads to a decrease in the total number of cells in culture. This decrease is apparently associated with a decrease in adhesion, especially at the early stages of exposure. In addition, the frequency of apoptosis in culture increases. Interestingly, the expression of stemness markers Oct4 and Nanog did not differ between the control and experimental groups, i.e., according to the authors, simulated microgravity did not increase the percentage of spontaneously differentiated cells [34].

However, the cultivation of mESCs in microgravity reduces their ability to form embryonic bodies [35]. Moreover, for mESCs in embryonic bodies, it was shown that a 15-day space flight (NASA STL experiment, STS-131) slows down differentiation while maintaining greater stemness [36]. Similarly, after clinostating, spontaneous neuronal differentiation was observed to slow down: a quantitative shift of cells towards early neuroblasts (less differentiated cells) was noted compared to the control [35], the beating of 10-day-old embryoid bodies decreased against the background of changes in the expression of genes involved in heart morphogenesis, as well as encoding proteins of the MAP kinase cascade, focal adhesion, and cytoskeleton [37]. Embryoid bodies formed by induced human pluripotent stem cells hiPSC were also characterized by the maintenance of greater stemness in simulated microgravity [38]. At the same time, in contrast to spontaneous differentiation, activin A-directed differentiation of mESCs into the definitive endoderm was enhanced under conditions of simulated microgravity [39].

Moreover, it is interesting that post-flight mESCs under 1 *g* conditions (that is, after the transition from 0 *g* to 1 *g*), on the contrary, were easier to differentiate into a colony of contracting cardiomyocytes, compared with the control group [36]. An increase in external mechanical stress to 50 *g*/5kPa hydrostatic pressure for 1 h led to the activation of the transcription of population maintenance and angiogenesis genes in mESCs. At the same time, the expression of genes associated with multicellular development, mechanotransduction, and DNA repair increased in embryoid bodies [40]. Additionally, the application of additional shear stress to human embryonic stem cells hESC line H1 led to an increase in H2B histone acetylation and an increase in the nucleus as a result of cytoskeletal reorganization [41].

### 2.3. Mesenchymal Stem Cells (MSC)

Significantly more often, the study of the effect of microgravity on cultured cells is carried out on mesenchymal stem cells than on ESCs. Moreover, in addition to real or simulated microgravity, a wide range of biophysical stimuli are used, such as the application of cyclic mechanical stress, fluid shear stress, changes in matrix stiffness, and substrate topography, which are perceived by stem cells [42]. Moreover, some of the stimuli may be interrelated and mediated by the MSC response.

It has been known for quite a long time that the rate of MSC division, their differentiation potential, and the cytoskeleton structure change under microgravity conditions [43,44,45,46,47], but, probably, each of these aspects significantly depends on the time spent in weightlessness.

It has been shown in a real space flight that 7-day cultivation of MSC does not lead to changes in the cell cycle; however, after 14 days, the expression of Polo-like kinase 1 (PLK1), a serine-threonine kinase involved in the cell cycle progression from the G2 phase to mitosis, decreases [48]. At the same time, the cells obtained after a 14-day flight and the cells of the control group did not show differences in response to the commitment to osteogenic or adipogenic differentiation; there were no changes in the integrity of chromatin. However, the secretion of some cytokines and growth factors changed in the flight group, while the immunosuppressive activity of flight MSCs increased compared to the control [48].

Many studies have noted changes in microfilaments, primarily the destruction of F-actin in cultured cells under microgravity conditions, which can lead to the activation of Rho-dependent signaling pathways [44,49,50,51]. Moreover, in MSCs, the expression of genes encoding actin and the actin-binding protein Arp2/3, as well as RhoA (a member of the GTPase Ras superfamily associated with the actin cytoskeleton), is reduced in microgravity [46,52,53,54,55].

However, RhoA activity varies depending on the duration of exposure of cultured cells under weightless conditions. For example, the cultivation of MSC rats in simulated microgravity for 72 h promoted endothelial, neuronal, and adipogenic differentiation against the background of a decrease in RhoA activity, and for 10 days, osteogenic differentiation with a significant increase in RhoA activity compared to control [56].

GTPase RhoA and the actomyosin cytoskeleton (but not the Hippo/LATS pathway) determine the transduction of mechanical signals from the extracellular matrix into the nucleus mediated by Yorkie-homologues YAP (Yes-associated protein) and TAZ (transcriptional coactivator WWTR1) [57]. Under conditions of simulated microgravity, the content of YAP in MSC nuclei decreases for 72 h [58], with a decrease in RhoA activity, as mentioned above [56]. Application of lysophosphohaditic acid after simulated microgravity increases the content of YAP in the nucleus, but the level of control is not achieved. At the same time, the use of low-intensity vibrations restores the content of YAP in the nucleus to the level of control, which gives grounds, according to the authors, to use this method as a countermeasure [58].

On the other hand, there is a direct mechanical link between the nucleus and the cytoplasm, mediated by the LINC complex (Linker of Nucleoskeleton and Cytoskeleton), which binds the structural proteins of the cytoplasm and the nucleus [59]. The giant transmembrane protein nesprin binds the actin cytoskeleton on the cytoplasmic side and the Sun protein complex on the nuclear side, which in turn anchors the network formed by lamin A/C. Since the translocation of YAP to the nucleus is blocked in cells with a deletion of nesprin-1, it can be assumed that mechanotransduction by YAP is a LINC-dependent process [60].

Additionally, the MAP-kinase cascade is associated with the actin cytoskeleton, and the level of phosphorylation of its participants, ERK1/2 and MAPK, decreases in simulated microgravity [49,61,62].

The microgravity reception, as well as hypergravity, can be mediated through a change in the cytoskeleton, but apparently in other ways leading to a different result. Thus, if under microgravity conditions, the spontaneous differentiation of MSCs from the rat bone marrow proceeds mainly in the adipogenic direction [63], then in hypergravity, it proceeds in the cardio- and osteogenic direction [63,64]. The same is true for MSCs isolated from adipose tissue: osteogenic differentiation increased under hypergravity conditions (up to 50 *g*), with exposure of 20–30 *g* having the greatest effect [65]. In another experiment, the culture of these cells was subjected to intermittent hypergravity at the level of 10 *g*, 20 *g*, 40 *g,* and 60 *g*: 3 times for 20 min with an interval of 40 min between exposures during which the cells were at 1 *g* [66]. The authors of the study showed that the modulus of elasticity, measured by atomic force microscopy, is reduced. In addition, the destruction of microfilaments is observed starting from 20 *g*, while a significant decrease in the number of microtubules starts from 40 *g* [66].

## 3. Somatic Cells

In differentiated single cells, microgravity conditions also cause a number of changes, primarily related to the structure of the cytoskeleton, metabolism, gene expression, and, in some cases, ion currents through ion channels in the membrane.

In U937 cells (human myelomonocytic cells) in parabolic flight and suborbital ballistic flights, in which there are conditions of both micro- and hypergravity, changes in the transcriptome were demonstrated as early as 20 s after gravity change. Moreover, only 2.4% of transcripts altered in microgravity were sensitive to the broad-spectrum cation channel inhibitor SFK-96365. At the same time, in microgravity, almost all (99.43%) of the altered transcripts were restored after 300 s, and in hypergravity (slightly less—98.93%), after 75 s. Interestingly, in both cases, the products of genes associated with intracellular transport, cell cycle, and cell division remained unreduced. However, in addition, the difference is that in microgravity, the transcripts of genes associated with mRNA processing and post-translational regulation gene expression were not restored, while under hypergravity conditions, those associated with metabolism and regulation of gene expression were at the transcription level [67].

Similar rapid changes in metabolic processes were noted in the NR8383 line of rat alveolar macrophages under microgravity conditions in the TRIPLELUXA experiment carried out aboard the International Space Station. The oxidative burst was assessed and showed its termination immediately upon reaching microgravity, recovery began already after 14 s, and the control level was reached after 42 s, which, according to the authors of the study, indicates a very rapid adaptation of macrophages to microgravity [68].

Changes in the cytoskeleton and cell shape under suborbital flight conditions are also observed at the earliest stages of exposure. In the FLUMIAS experiment in primary human macrophages during the TEXUS-54 suborbital flight, the state of the nucleus, cytoplasm, lysosomes, and actin cytoskeleton was assessed. A change in the shape of cells under microgravity conditions was shown: after 126–151 s of flight, the volume of the cells as a whole and the nucleus were increased relative to the control, while after 201–226 s, the volume and surface area of both the cells as a whole and the nucleus decreased relative to the control. At the same time, actin fluorescence decreased even earlier, after 4–19 s of microgravity, and recovered after 126–151 s [69].

Following the transient changes in gene expression, metabolism, and cytoskeleton, which were observed in the first seconds of exposure to altered gravitational conditions, after several tens of minutes, more stable changes occurred, to which, nevertheless, an adaptive structural and functional pattern was also formed.

In mouse chondrocyte progenitors (ATDC5 cell line), a decrease in c-fos expression was noted as early as 30 min and 60 min after exposure to 32 *g* conditions. Moreover, if the cultivation was carried out for 3 days with daily exposure for 1 h at 32 *g*, c-fos mRNA was also reduced, although the content of mRNA of another member of the AP-1 transcription complex, c-jun, remained at the control level. In addition, the authors observed some decrease in actin filament polymerization after 1 h of exposure to 18.7 *g*. Using an inhibitory assay, a decrease in actin polymerization in hypergravity via ROCK/Rho-GTP and PI3K was shown to be involved in the down-regulation of c-fos [70].

In human chondrocytes exposed to a random positioning machine (simulated microgravity), after 30 min, an increase in the expression of genes whose products are involved in cell growth and differentiation, as well as various structures of the cytoskeleton—beta-actin, beta-tubulin, and vimentin, was noted. However, after 4 h, the network of intermediate filaments was destroyed, although after 16 h, the changes were not so dramatic—the cytoskeleton was reorganized [71].

Hypergravity at 5 *g*, 10 *g*, 15 *g,* and 20 *g* of similar duration (4 and 16 h) in human tendon cells (hTDC) resulted in reduced cell proliferation and increased cell area, especially after 16 h. Additionally, after 16 h, cells cultured at 5 *g* and 10 *g* showed an increase in actin filaments. The content of focal adhesion kinase (FAK) increased with an increase in the level of hypergravity, and its localization shifted towards focal adhesion sites, in contrast to the perinuclear localization in the control [72].

Significantly longer cultivation in simulated microgravity of human renal epithelial cells (line ARPE-19) led to the almost complete disappearance of F-actin after 5 days, remaining only in the area under the cell membrane. However, after 10 days, the distribution of F-actin was the same as in the control. However, the content of beta-actin decreased after 5 days, and after 10 days, the expression of the gene encoding it decreased. The content of beta-tubulin did not change, but the expression decreased after 10 days. The content of the protein of intermediate filaments—vimentin— increased after 5 days, and after 10 days, decreased against the background of a decrease in the expression of the gene encoding it. The expression of keratin Krt8, lamin LmnB2, and fibronectin also decreased. According to the authors of the study, the above changes may be associated with the VEGF-dependent pathway—VEGF expression also decreased after 5 days of simulated microgravity [73].

## 4. Gamets

There are few studies of germ cells, spermatozoa, and oocytes—which in most species are free single cells—under conditions of weightlessness, real or simulated. Changes in conditions of micro- or hypergravity of reproductive tissues, in which gametes mature, have a significant effect on spermatozoa and oocytes. However, there are practically no data on the cultivation of germ cells directly under micro- or hypergravity conditions.

### 4.1. Spermatozoa

Under space flight conditions and in model experiments, the number of mature spermatozoa in the epididymis decreases, and in simulated weightlessness, the decrease is more pronounced [74,75,76,77]. Moreover, the number of progenitor cells, spermatogonia, decreases in the seminiferous tubules of rats after 13–14 daily space flights of the KOSMOS 1887 [78] and KOSMOS 2044 satellites [77]. According to radiation dosimetry data, this reduction effect is not fully associated with ionizing radiation [78]. On the other hand, a decrease in testosterone concentration is observed with a short exposure to weightlessness [79,80], but this effect becomes significant when the hormone concentration decreases by more than 70% [81]. However, antiorthostatic suspension of B6D2F1 mice for 7 days resulted in a less dramatic decrease in serum testosterone levels but disrupted spermatogenesis. The proportion of motile spermatozoa did not differ between groups, but the average speed of movement was statistically significantly reduced in suspended mice [82].

After 30 days of antiorthostatic suspension of mice, we observed various changes in their spermogram: a significant decrease in the number of motile spermatozoa and in the proportion of viable and normal spermatozoa. According to the data on the content of sperm-specific proteins, there was a shift towards immature forms, as well as various changes in the content of cytoskeletal proteins and the expression of the corresponding genes [83]. A 30-day unloading did not lead to a change in the overall level of DNA methylation; however, after a 12 h recovery, its decrease was observed, which may indicate a probable increase in gene expression in the early period of readaptation. In addition, the relative content of HAT1 acetylase increased in the group where there was a change in the level of DNA methylation, which possibly led to an increase in the level of histone acetylation. The latter, in turn, can regulate the efficiency of transcription both directly by reducing histone binding to DNA due to charge changes [84] and by triggering a long chain, the participants of which can include chromatin remodeling proteins, transcription activators, proteins that bind to methylated DNA, and recruiting methylases.

In longer experiments (6-week antiorthostatic suspension of rats), the concentration of testosterone, luteinizing hormone (LH), and follicle-stimulating hormone (FSH) did not change, but testis mass and spermatogenesis were reduced; no spermatogenic cells were observed, with the exception of a few spermatids; and, accordingly, mature sperm in the epididymis was not [85]. Similarly, after a 91-day spaceflight, LH and FSH receptor expression did not change in male C57BL/10J mice (one wild-type and two pleiotrophin overexpressing mice), but epididymal sperm count was reduced by about 90% compared to controls [86].

In the Rodent Research 4 experiment (SpaceX-10 mission, February 2017, USA), tissue sampling took place under weightless conditions on the ISS, which excluded the impact of factors such as landing overload and the recovery period during transportation to the laboratory. The data obtained showed that the content of cytoskeletal proteins in ductus deferens did not change, but there was a decrease in the expression of genes encoding beta-tubulin and alpha-actinin-1. In addition, we found an increase in the expression of the gene encoding a protein specific for spermatogonia Kdm5b (Jarid1B), a decrease in the S-phase methylase transcript Dnmt1, and an increase in the demethylase of the TET protein family Tet2, as well as a decrease in the relative content of Hdac1 histone deacetylase mRNA and an increase in acetylase Hat1. These changes indicate a shift towards less-differentiated forms and epigenetic events that may regulate the formation of an adaptive pattern [87].

In another study, 12 male mice were divided into 2 groups (real microgravity and centrifugation to create 1 *g*) and kept aboard the ISS for 35 days. In males of both flight groups, a decrease in the mass of the prostate gland was found; however, none of the reproductive organs had any obvious microscopic defects [88]. Motility parameters of spermatozoa located in the caudal epididymis of mice of the flight groups decreased 2 h after incubation, but this did not affect the ability to fertilize oocytes in vitro. The growth rates and fertility of the resulting pups were comparable in all study groups. In addition, gene expression was analyzed using RNA-seq, and the authors did not find any changes [88]. The difference from the data of the RR-4 experiment is primarily due to the fact that in this experiment, the mice were brought to Earth alive, and tissue sampling took place only 2 days after exposure to microgravity conditions, while in the RR-4 experiment, samples were collected in space [87].

Therefore, it can be assumed that after 12 h of readaptation after a 30-day suspension, the recovery process has not yet been completed, but a 2-day stay under the Earth’s gravity after a 35-day space flight leads to an almost complete restoration of the reproductive function of male mice.

However, although storage of mouse spermatozoa at −90 °C on the ISS, where the radiation background is 100 times higher than the ground level, for 9 months led to an increase in DNA damage, nevertheless, fertilization and birth were normal, although with some decrease compared to the control group. Next-generation sequencing showed only minor genomic differences between progeny derived from space-preserved spermatozoa and controls, and all progeny grew to maturity and had normal fertility [89].

Human sperm samples were exposed on a clinostat and in parabolic flight, in which there were 10 cycles of alternating 2 *g* hypergravity (25 s) and then microgravity (20 s). Under conditions of simulated microgravity, a tendency of a decrease in the speed of movement was noted; in parabolic flight, spermatozoa motility significantly decreased in microgravity and even more significantly in hypergravity [90].

Slightly longer exposure of mouse spermatozoa in simulated microgravity (for 6 h) leads to a significant decrease in motility, but under 2 *g* conditions, such changes are observed after 1 h, against the background of changes in the content of cytoskeletal proteins [91], which is quite consistent with the results of previous studies [90]. However, there is evidence that bull spermatozoa demonstrate an improvement in motility parameters under the influence of microgravity within 6 min, which may indicate the possible existence of an ambiguous gravitational response of mammalian spermatozoa [92].

However, the spermatozoa of lower animals exhibit different responses to changes in the force of gravity acting on them.

A detailed study of the motor activity of sea urchin spermatozoa under space flight conditions was carried out by the group of Tash J.S. with co-authors. Spermatozoa were isolated from sea urchins and, prior to the experiment, were stored in a buffer that maintained their immobility at a temperature of 4–5 °C until the start of the experiment under space flight conditions. Approximately 19 h after the start, the cassettes with spermatozoa were heated to 22 °C, the spermatozoa were activated with seawater, and an increase in motility was noted immediately, 30 and 60 s after the start of activation. Then, the cassettes were fixed and stored at −20 °C until they were returned to Earth, where the protein content was studied. The sea urchin FP130 protein, whose phosphorylation changed most clearly under microgravity conditions, was characterized as the I1 subunit of the inner handle of dynein and is homologous to the *Chlamydomonas* FP138 protein, the regulation of which is carried out by phosphorylation and dephosphorylation through the kinase/phosphatase system [93,94,95,96]. Its phosphorylation, which is observed to increase in microgravity, is also observed in hypergravity during the first 30 s from the start of the experiment using an onboard centrifuge and a NiZeMi microscope. Moreover, under hypergravity conditions, FP130 phosphorylation does not decrease with the addition of H89, an inhibitor of protein kinase A, which suggests that changes in phosphatase activity play a leading role in the gravitational response of sea urchin spermatozoa [97]. Under conditions of simulated microgravity, the speed of movement of *Drosophila melanogaster* spermatozoa also increases after 6 h of exposure to a random positioning machine [98].

To reveal the mechanism of different responses of mammalian and lower animal spermatozoa to microgravity conditions, we performed a motility analysis using kinase and phosphatase inhibitors. We have shown that an increase in the speed of movement of fly spermatozoa is prevented by the administration of an inhibitor of Ser/Thr phosphatase, which may indicate the activation of additional phosphatase activity under simulated microgravity. In mouse spermatozoa, after 6 h of simulated microgravity, the decrease in motor activity is blocked by a kinase inhibitor. Moreover, it is interesting that the introduction of phosphatase inhibitors before exposure to simulated microgravity prevents not only the effects of changes in the rate but also in the content of actin-binding proteins [99].

This was confirmed by data on the motility of spermatozoa of the fruit fly *Drosophila melanogaster* after a 12-day space flight [100]. Due to a number of technical problems, it is not possible to conduct lifetime experiments at the landing site. Therefore, the study of motor activity was carried out only in laboratory conditions (16 h after landing), and we saw that the speed in the flight group was significantly (*p* < 0.05) lower than the control level by 20%. We assumed that landing overloads and the 16 h readaptation period play the leading role in this effect. Therefore, in order to analyze the role of overloads, weightlessness, and the early period of readaptation, a simulation experiment was carried out synchronously to space flight using equipment that allows reproducing the cyclogram of overloads during launch and landing and weightlessness. The results obtained indicate that under conditions of simulated weightlessness, the speed increases, and overloads during landing destroy this effect, reducing the speed to the level of control. The early readaptation period leads to an even greater decrease in speed, and thus the values that we observed in the space flight group are obtained. In other words, landing overload and readaptation lead to a decrease in the motor activity of fly spermatozoa, and this decrease is not associated with a change in the energy supply of motor activity—the rate of oxygen uptake, the content of respiratory chain proteins, and their gene expression remain unchanged. Using a broad-spectrum kinase inhibitor 6-(dimethylamino)purine (6-DMAP) and phosphatase inhibitors (tyrosine-sodium orthovanadate, serine-threonine-calyculin A), it was shown that an increase in the speed of movement under weightless conditions occurs as a result of additional protein kinase activity. Such protein kinases, whose activity in spermatozoa leads to an increase in the speed of movement, can be, for example, cAMP-dependent protein kinase A (PKA) and protein kinase C (PKC), the activity of the latter being inhibited by the 6-DMAP analog staurosporine. Under the action of overloads and in the early period of readaptation, the activity of other protein kinases that suppress motor activity and phosphatases increases against the background of a decrease in the content of microtubule proteins. Such protein kinases, the action of which will lead to a decrease in the motor activity of spermatozoa, can be, for example, glycogen synthase kinase 3 (GSK3) and phosphatidylinositol 3-kinase (PI3K). It should be noted, as the main result of this experiment, that the use of 6-DMAP makes it possible to restore the reduced speed of spermatozoa in the flight group to the control level [100].

On the other hand, such an insignificant selection of the results of studies presented in the literature to date, the duration and organization of the experiment of which are not comparable, does not allow us to draw unambiguous conclusions about the regulation of the motor activity of spermatozoa in various animals under conditions of real or simulated microgravity.

### 4.2. Oocyte

The antiorthostatic suspension model can also be a good model for the analysis of reproductive processes in female mice when modeling the effects of microgravity [101]. Thus, when using this model for the 23-day gravitational unloading of female BALB/c mice, we observed slight changes in the protein composition of the cytoskeleton against the background of an increase in the mRNA content of the corresponding genes. It can be assumed that the regulation of the protein pattern occurs primarily at the level of translation. At the same time, the increase in expression is probably due to the establishment of a hypomethylated state of DNA. The latter seems to be due to active complete demethylation of the target sites without accumulation of the 5hmC intermediate [102].

In the ovaries of flies, during the complete cycle of oogenesis, which took place under conditions of simulated microgravity, we found an increase in cellular respiration, and, according to inhibitory analysis, apparently due to complex II of the respiratory chain against the background of a constant content of proteins of the respiratory chain and the area of oocytes in ovarioles [103]. We associated these changes with the observed decrease in the content of the actin-binding protein alpha-actinin and beta-actin, which in turn can activate the transcription factor STAT3 [104], which can activate cellular respiration due to the activation of complexes I and II of the mitochondrial respiratory chain [105,106,107]. Moreover, the introduction of essential phospholipids prevents all changes caused by microgravity [103]. However, if flies from a zygote to a 2-day-old individual developed under conditions of simulated microgravity, we also observed an increase in the intensity of cellular respiration, but at the expense of complex I of the respiratory chain. Moreover, the content of cytoskeletal proteins, actin, and alpha-actinin, on the contrary, increased [108]. This increase is probably due to an increase in transcription efficiency, which correlates with an increase in the proportion of histone H3 acetylated at Lys9 [109].

In the case of exposure to microgravity conditions of follicles or oocytes, numerous structural changes are also observed.

Cultivation in simulated weightlessness of mouse preantral follicles in vitro revealed disruption of microvilli in the *zona pellucida*, the presence of vacuolized mitochondria and cytoplasmic vacuoles [110], and a decrease in follicle survival, probably due to aberrant development of granulosa cells [111]. In another experiment, Kunming mouse oocytes were harvested at the germinal vesicle stage (which is the metaphase of the first meiotic division) by superovulation stimulation. Cultivation of oocytes in simulated microgravity for 6, 9, and 16 h led to a dramatic decrease in the number of oocytes (by eight times) that reached the metaphase of the second meiotic division (estimated by the extrusion of the first polar body), and blebbing appeared. The authors associated the observed changes with abnormal formation of the meiotic spindle during both the first and second meiotic divisions as a result of abnormal localization of γ-tubulin. At the same time, the authors did not note changes in microfilaments [112].

Thus, it seems quite interesting that the results of studies of cultured cells and single cells with different degrees of differentiation do not contradict the results of studies of the structure and function of cells in tissues in experiments on animals in vivo. Changes in the structure of the cytoskeleton; changes in cellular metabolism, gene expression, and its regulation at different levels; the cell cycle; and the differentiation potential are noted both for single cells and cells in tissues under conditions of micro- and hypergravity. For different types of cells, different time dependences of the development of the physiological response are observed. In other words, a change in gravity affects even a single cell, leading to a change in all intracellular processes.

## 5. Mechanoreception and Mechanotransduction in the Cell

The cell is formed in the field of constant gravity. Accordingly, all intracellular structures must have such mechanical characteristics in order to maintain the shape and volume of the cell in this field. Additionally, the speed and direction of metabolic processes should lead to the production of a sufficient amount of macroergs to carry out all the processes of the cell’s vital activity in this field. In other words, the structural and functional capabilities of the cell are “tuned” precisely to the external mechanical field in which this cell was formed.

However, the key question remains how the same cell perceives opposite changes in the external field, microgravity, and hypergravity. In other words, the primary sensor of the external gravitational field must have three modalities depending on the acting gravity:

“E” state—the gravity in which this cell was formed;

“E+” state—hypergravity relative to the initial state (if the cell was formed, for example, in weightlessness, then the gravity of the Earth will be hypergravity in this case; for a cell formed under the gravity of the Earth, transfer to a centrifuge with a rotation of more than 1 *g* will be hypergravity);

“E−” state—state of weightlessness or microgravity relative to the initial state (for a cell that appeared under the conditions of the Earth’s gravity, weightlessness will be microgravity; for a cell formed, for example, when rotating on a centrifuge in conditions greater than 1 *g*, stopping the centrifuge will mean a transition to “microgravity” conditions).

Such conditions imposed on the sensor limit its choice. It seems quite obvious, from a biophysical point of view, that any change in the field of an external force, in particular, the force of gravity acting on the cell, will lead to the occurrence of a mechanical deformation [113]. Indeed, during the suborbital flight of TEXUS-54, direct evidence of the onset of deformation was experimentally obtained—a change in the shape of the cells was recorded after a few seconds [69].

From a physical point of view, it is clear that the amount of deformation of an object depends on the force applied to it and the object’s own mechanical characteristics. The intrinsic mechanical properties of cells are determined primarily by cytoskeletal structures. For cells of higher animals with a developed cytoskeletal network, the concept of tensegrity is of great interest [114]. However, in the evolutionary series, not all cells have a developed cytoskeleton that permeates the entire cell, but all cells have a membrane and a cortical cytoskeleton associated with it. This barrier was the first in evolution that separated the intracellular contents from the external environment and allowed maintaining the homeostasis of the volume and shape of the cell.

Therefore, it is important to consider the cortical cytoskeleton and the membrane associated with it as the primary mechanosensor. Such a mechanosensor can exist in three states: undeformed state, tensile deformation, or compressive deformation with an increase or decrease in external mechanical stress; for example, with a change in gravity. It should be noted that we are talking only about the very first moments of changes in external mechanical stress.

We have measured the stiffness of various cells and, based on them, proposed a mathematical model and calculated the magnitude of the deformation when the force of gravity changes by 1 *g* (for example, from 1 *g* to 0 *g,* or in the opposite direction) [113,115,116,117,118,119]. The results of mathematical modeling and comparison with experimental data indicate that such deformation energy is sufficient for dissociation, for example, from actin filaments of actin-binding proteins.

**Figure 1:**
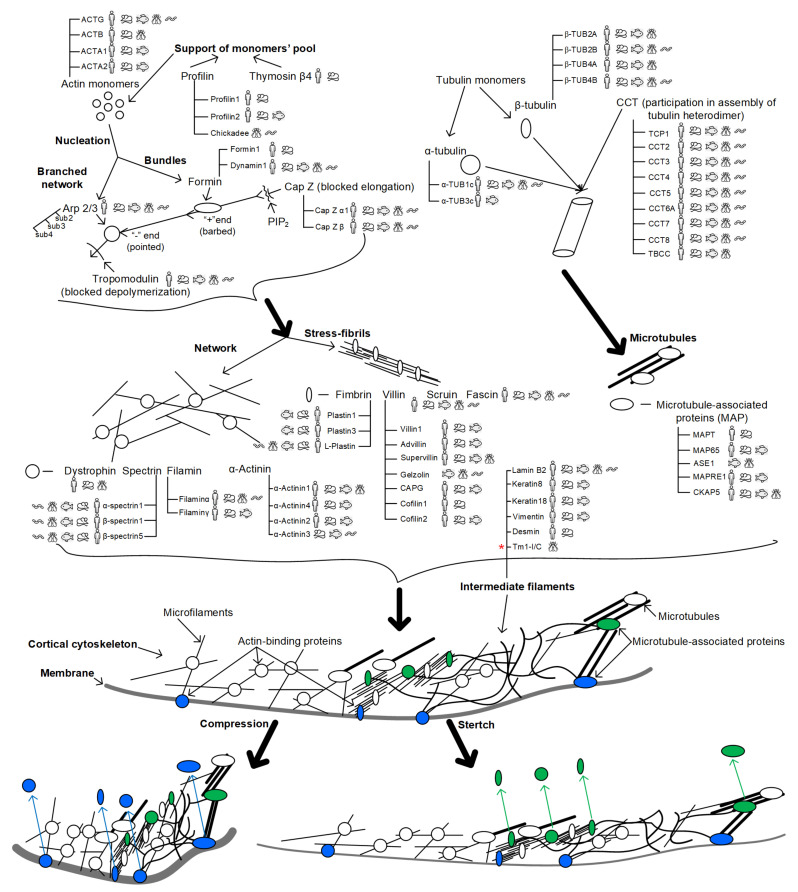
Possible scheme of cellular mechanoreception. To demonstrate the variability of possible participants in mechanoreception, the main proteins involved in the organization of the components of the cytoskeleton are presented. Pictograms indicate the expression of these genes in various animal species—*H. sapiens*, *M. musculus*, *D. rerio*, *D. melanogaster*, and *C. elegans* (according to the open resource HomoloGene https://www.ncbi.nlm.nih.gov/homologene, accessed on 21 September 2022). Microfilaments: the pool of actin monomers is maintained by profilin family proteins and thymosin β4; monomers polymerize into filaments, and their length is controlled by tropomodulin (at the pointed end) and CapZ (at the barbed end); microfilaments either stack in bundles (with formin nucleation) or form a branched network (with Arp 2/3 nucleation); a network of microfilaments and stress fibrils is organized by actin-binding proteins. Microtubules: tubulin monomers, alpha- and beta-, form a heterodimer with the participation of proteins of the CCT family; heterodimers are assembled into microtubules, the spatial organization of which and association with other intracellular structures is carried out by MAP proteins. Intermediate filaments: due to the presence of rod-like domains in the monomers, intermediate filaments are assembled, which can be localized in the nucleus (lamins) and in the cytoplasm. Not so long ago, it was believed that *Drosophila* lacks cytoplasmic intermediate filaments [124] and that the cell structure is strengthened at the expense of other components of the cytoskeleton. Therefore, it seems important to note (by red asterisk) recent data indicating that *D. melanogaster* has cytoplasmic intermediate filaments formed by the Tm1-I/C protein [125,126]. A change in external mechanical stress (for example, gravity) will lead to deformation. Compressive deformation would possibly lead to dissociation from the cortical cytoskeleton of the proteins anchoring it to the membrane—these are highlighted in green. Tensile deformation may lead to dissociation from the cortical cytoskeleton of proteins that organize the parallel stacked components of the cytoskeleton—they are highlighted in blue. Highlighted proteins diffuse from the cortical cytoskeleton under tension and contraction, as indicated by colored arrows. In both cases, the choice of specific participants in the process can be species-specific.

Because the cortical cytoskeleton is heterogeneous, tensile and compressive strains can lead to the dissociation of various proteins (Figure 1). For example, when stretched, proteins that form the longitudinal structures of the cortical cytoskeleton can dissociate; during compression deformation, proteins anchoring the cytoskeleton to the membrane can dissociate.

A systematic analysis of possible gravireceptors in eukaryotes indicates different ways of responding to changes in the gravitational stimulus, but almost always mediated by the cytoskeleton [120]. It should be noted that, in evolution, orthologs can belong to different cytoskeletal structures, which, accordingly, can determine a wide range of possible gravireceptors in different species. For example, in experiments on mammals, we have shown that in the case of micro- and hypergravity, various actin-binding proteins can dissociate from the cortical cytoskeleton: alpha-actinin1 and alpha-actinin4, leading to the launch of various pathways and, further, to various changes in gene expression [15,16,121]. Moreover, the experimental increase in the stiffness of the cortical cytoskeleton of cells in experiments on rodents in vivo makes it possible to prevent the dissociation of actin-binding proteins and, as a result, prevent atrophic changes in muscle cells and negative changes of spermogram parameters during antiorthostatic suspension [83,122]. However, in *Drosophila*, there is only one alpha-actinin isoform; therefore, the actin-binding protein fimbrin can also be the second protein [123]. In addition, the role of non-coding RNAs cannot be excluded, which can bind various cytoskeletal structures, for example, alpha-actinin4 and beta-tubulin [32], and, accordingly, when their localization changes, they can trigger the underlying signaling pathways in some cells.

**Figure 2:**
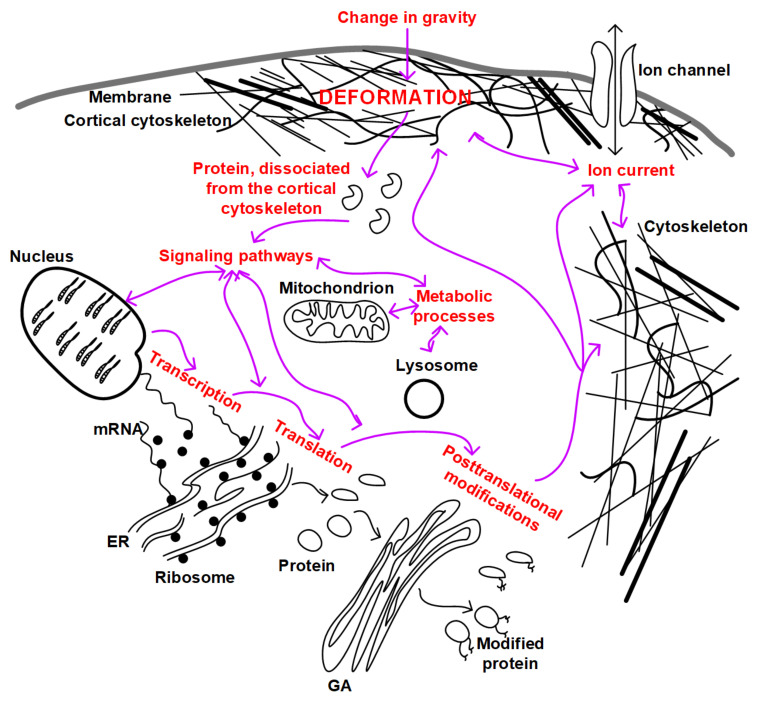
Relationship of mechanotransduction pathways in a single cell. The main cellular structures are schematically indicated: membrane, cytoskeleton, nucleus, endoplasmic reticulum (ER) and ribosomes, Golgi apparatus (GA), mitochondrion, and lysosome. Cytoskeletal structures penetrate the cell through and through and connect all organelles to each other, forming a cytoskeletal network. The red labels indicate the main processes that can be targeted as a result of gravity change transduction. Purple arrows indicate possible mutual regulation of intracellular processes.

Summarizing all of the above, we can propose an integrated scheme for the development and relationship of mechanotransduction events in the cell (Figure 2). If we assume that as a result of micro- and hypergravity, different deformations occur due to the heterogeneity of the structure of the cortical cytoskeleton, then, as a result, different proteins dissociate. These proteins can themselves play a signaling role and can interact with participants in other signaling pathways, leading to changes in the expression of target genes, regulation of translation and post-translational modifications, and metabolic processes in the cell. In addition, a possible change in the structure of the cytoskeleton can lead to a change in the conductivity of ion channels, the binding affinity of signaling molecules, and a change in the localization of intracellular structures (for example, the nucleus or mitochondria). All this taken together will lead to the formation of an appropriate adaptive structural and functional pattern of cells.

## 6. Conclusions and Perspectives

The concept of evo-devo implies, among other things, considering the various stages of development in an evolutionary aspect. The gravitational field accompanies the development of the biosphere throughout its evolution and the development of an individual organism throughout its ontogenesis. Therefore, it can be assumed that the reception of gravity is one of the most evolutionarily ancient mechanisms inherent in all living organisms, regardless of the level of organization. Since the membrane with some kind of supporting structure separated some contents from the external environment, essentially forming the first cell, one could assume its participation in the reception of gravity. Subsequent evolution led to variability in the lipid composition of the membrane and components of the cortical cytoskeleton, but apparently, retained this structure’s trigger role in the transduction of changes in the force of gravity acting on the cell. The concept proposed above and an equal mechanism based on the dissociation of various structural elements (in particular, proteins, but other variants are also possible) from the cortical cytoskeleton can explain the difference in the adaptive pattern of cells under micro- and hypergravity conditions, as well as the variability of possible gravireceptors on the molecular levels in different cells of different species.

This review presents, first of all, the data concerning single cells from the perspective of the development of a new organism under conditions of altered gravity—germ cells and preimplantation embryos. The data concerning cultured cells and, moreover, some differentiated cells are presented in order to show the role of the cytoskeleton in gravireception. Data concerning experiments on malignant cells are not presented at all since this is a separate area that requires each time a detailed analysis of the cytoskeletal structures of one or another line of cancer cells. However, it seems that such an analysis should certainly be made in future studies since changes in the cytoskeleton in cancer cells may be of exceptional interest for analyzing the role of its individual components in gravireception. In addition, studies of cells of evolutionarily distant organisms, including unicellular eukaryotes, will be able to provide more insight into the reception of the gravitational field by individual cells. In turn, the discovery of the mechanism of gravity reception and transduction at the cellular level will make it possible to develop fundamentally new approaches to the protection of the human body in conditions of long-term space flight, including the exploration of other bodies of the solar system and deep space.

## Data Availability

All data generated or analyzed during this study are included in this article.
